# Evolution of SARS-CoV-2 Envelope, Membrane, Nucleocapsid, and Spike Structural Proteins from the Beginning of the Pandemic to September 2020: A Global and Regional Approach by Epidemiological Week

**DOI:** 10.3390/v13020243

**Published:** 2021-02-04

**Authors:** Paloma Troyano-Hernáez, Roberto Reinosa, África Holguín

**Affiliations:** HIV-1 Molecular Epidemiology Laboratory, Microbiology and Parasitology Department and Instituto Ramón y Cajal para la Investigación Sanitaria (IRYCIS), Hospital Universitario Ramón y Cajal, CIBER en Epidemiología y Salud Pública (CIBERESP), Red en Investigación Translacional en Infecciones Pediátricas (RITIP), M-607, km. 9, 100, 28034 Madrid, Spain; troyanopaloma@gmail.com (P.T.-H.); roberto117343@gmail.com (R.R.)

**Keywords:** SARS-CoV-2, spike, nucleocapsid, envelope, membrane, D614G, R203K, G204R, genetic variability, structural proteins

## Abstract

Monitoring acute respiratory syndrome coronavirus 2 (SARS-CoV-2) genetic diversity and emerging mutations in this ongoing pandemic is crucial for understanding its evolution and assuring the performance of diagnostic tests, vaccines, and therapies against coronavirus disease (COVID-19). This study reports on the amino acid (aa) conservation degree and the global and regional temporal evolution by epidemiological week for each residue of the following four structural SARS-CoV-2 proteins: spike, envelope, membrane, and nucleocapsid. All, 105,276 worldwide SARS-CoV-2 complete and partial sequences from 117 countries available in the Global Initiative on Sharing All Influenza Data (GISAID) from 29 December 2019 to 12 September 2020 were downloaded and processed using an in-house bioinformatics tool. Despite the extremely high conservation of SARS-CoV-2 structural proteins (>99%), all presented aa changes, i.e., 142 aa changes in 65 of the 75 envelope aa, 291 aa changes in 165 of the 222 membrane aa, 890 aa changes in 359 of the 419 nucleocapsid aa, and 2671 changes in 1132 of the 1273 spike aa. Mutations evolution differed across geographic regions and epidemiological weeks (epiweeks). The most prevalent aa changes were D614G (81.5%) in the spike protein, followed by the R203K and G204R combination (37%) in the nucleocapsid protein. The presented data provide insight into the genetic variability of SARS-CoV-2 structural proteins during the pandemic and highlights local and worldwide emerging aa changes of interest for further SARS-CoV-2 structural and functional analysis.

## 1. Introduction

Coronavirus disease (COVID-19) was detected for the first time, in Wuhan, China, in December 2019 [1]. In January 2020, the responsible virus, acute respiratory syndrome coronavirus 2 (SARS-CoV-2) was isolated and the complete viral genome was sequenced [2]. Since then, SARS-CoV-2 has spread throughout the world in this ongoing pandemic. SARS-CoV-2 is a ß-coronavirus belonging to the Coronaviridae family, order Nidovirales. Coronaviruses (CoVs) are enveloped positive-sense RNA viruses with a large and non-segmented genome of ∼30 kb length including five major open reading frames (ORFs), i.e., ORF1a, ORF1ab, and the four structural proteins plus a number of accessory genes [3]. The first two overlapping ORFs (ORF1a and ORF1b) are located at the 5′ end of the viral RNA, occupying about two-thirds of the genome, and encode proteins which are auto-proteolytically processed into 16 non-structural proteins (nsp), which are involved in viral RNA replication and transcription [4,5]. Interactions among several SARS-CoV-2 non-structural and human proteins have been highlighted [6]. The 3′ end of the viral genome encodes four main structural proteins, i.e., spike (S), envelope (E), membrane (M), and nucleocapsid (N), all required for the structurally complete viral particle [4].

The coronaviruses S protein is a trimeric glycoprotein that belongs to class I fusion proteins containing two subunits (S1 and S2), mediating attachment and fusion of viral and cellular membranes, respectively [7,8]. It allows viral entry by attachment of the S1 subunit to the host cell’s receptor angiotensin-converting enzyme 2 (ACE2) through the S1 receptor-binding domain (RBD), while the S2 subunit allows virus-cell fusion of viral and cellular membranes [8,9]. This process requires S protein priming by host proteases such as TMPRSS2 in cleavage sites S1/S2, a polybasic (furin) cleavage motif, at the S1/S2 boundary, and the S2′ site [8,9,10].

The E protein is the less abundant protein in the virion, but essential for correct virus assembly [11] and particle release through interaction with the M protein [12,13]. This protein is involved in critical aspects of the viral life cycle and CoVs lacking the E protein make promising vaccine candidates [13].

The M CoVs protein is a small, functionally dimeric protein, with three transmembrane domains that can adopt two different conformations [14]. It is the most abundant structural protein in the virion and plays a major role in assembly, participating in E assembly and N attachment to the viral genome [15]. Its membrane-altering properties rely on the interaction with other viral components such as N, S, and viral RNA [14].

The N protein has two main domains (N-terminal and C-terminal) that can bind to SARS-CoV-2 RNA forming the long, flexible, helical viral nucleocapsid [16] and interacts with the M protein during viral assembly [14]. It is considered to be a multifunctional protein since it is required for optimal SARS-CoV-2 replication, it enhances the efficiency of virus transcription and assembly, and it plays an important role in viral pathogenesis, triggering the host response to viral infection [17]. The viral nucleocapsid (RNA + N protein) is synthesized in the cytoplasm, whereas the other structural proteins, i.e., S, M, and E proteins, are transcribed and translated in the endoplasmatic reticulum and transported to the Golgi apparatus [4].

The SARS-CoV-2 genome presents a high homology to other human and bat CoVs, varying across genomic regions [2,18,19]. Recent studies have shown that SARS-CoV-2 shares around 89% sequence identity with other CoVs [20], showing the greatest homology with related bat-derived CoVs (88%) and less similarity with SARS-CoV (79%) or MERS-CoV (50%) [21].

Although RNA viruses have mutation rates up to a million times higher than their hosts correlated with enhanced virulence and viral evolution capacity [22], CoVs have genetic proofreading mechanisms absent in other RNA viruses, which limit their mutation rate [23]. The CoVs RNA-dependent RNA polymerase (RdRp), encoded by nsp12, plays a central role in both viral RNA replication and transcription [22]. RdRp associates with other viral non-structural proteins, forming a highly active and processive RNA polymerase complex [23]. Among these non-structural proteins, nsp14 presents an RNA3′–5′ exoribonuclease involved in viral RNA proofreading activities [23,24].

Despite their limited genomic diversity [25], with an estimated mutation rate lower than other RNA viruses, around 6 × 10^−4^ nucleotides/genome/year [18], SARS-CoV-2 presents mutations along its genome, including deletions on coding and non-coding regions [26]. CoVs can also recombine through homologous and nonhomologous recombination [27], which may be related to CoVs’ ability for interspecies jumping [28]. Furthermore, it has been demonstrated that a minimal variation in the SARS-CoV-2 genome may be responsible for a drastic change in the structures of drug target proteins, which would make some available drugs ineffective [20]. Therefore, it is essential to monitor SARS-CoV-2 genetic variability in this ongoing pandemic. This knowledge is crucial in order to understand its evolution and assure the performance of developing diagnostic tools, vaccines, and immunotherapeutic interventions against COVID-19.

This study aims to perform a descriptive analysis reporting on the degree of conservation of the four main structural SARS-CoV-2 proteins in the largest set of SARS-CoV-2 worldwide sequences collected from the Global Initiative on Sharing All Influenza Data (GISAID) from December 2019 to 19 September 2020. For this purpose, we used an in-house bioinformatics tool developed in our laboratory designed for genetic variability analysis of pathogens and proteins with biological or biomedical interest. We identified the most prevalent aa changes at a global, regional, and local level, and highlighted any changes with an increasing frequency in time or located in protein regions of special biological or structural interest.

## 2. Materials and Methods

All available complete and partial SARS-CoV-2 human genomic sequences deposited in the GISAID database (https://www.gisaid.org/) until 19 September 2020 were downloaded in nucleotides (nt) and classified according to the country of origin and to the epidemiological week (epiweek) by collection date. Epiweeks are a standardized method of counting weeks to allow for the comparison of data. By definition, the first epiweek of the year ends on the first Saturday of January, as long as it falls at least four days into the month. Each epiweek begins on a Sunday and ends on a Saturday. The analyzed SARS-CoV-2 sequences were deposited in GISAID from 29 December 2019 (epiweek 52, 2019) to 19 September 2020 (epiweeks 1 to 37, 2020).

For sequences analysis, we used an in-house bioinformatics tool previously designed and used in our laboratory for HIV genetic variability analysis and recently updated for SARS-CoV-2 sequences study [29,30,31,32]. This tool is programmed in JAVA OpenJDK version 11.0.9.1 using IDE NetBeans version 12.2. Functions related to protein tracking, cutting, and aligning were tested with Mega X, and functions related to aa change identification were tested manually, using Excel 2019 version 19.0. With this program, the complete nt sequences from the four structural viral proteins were cut, aligned, and translated into the following amino acids (aa): spike or S (1273 aa), nucleocapsid or N (419 aa), membrane or M (222 aa), and envelope or E (75 aa). Wuhan SARS-CoV-2 was taken as the reference sequence (NCBI accession number NC 045512.2) to identify the aa changes in these proteins. The program detects any aa different from the reference sequence for each aa position and calculates the number and frequency of aa changes for that position. Nonsense mutations, gaps, and unknown amino acids (probably due to the low quality of some regions of the original sequences, failing to attribute a nucleotide with certainty) were not considered to calculate the mutation rate. This method allows the analysis of partial or low-quality genomes as long as the residue of the studied position is present enabling a much larger set of sequences to be studied. The statistical average of the aa changes was comparatively analyzed between epiweeks and regions.

To detect any emerging aa change, sequences were analyzed all together (global analysis) and by geographic region (regional analysis), establishing the following six regions of interest according to GISAID classification: Africa, Asia, Europe, North America (including Central America and the Caribbean), South America, and Oceania. Sequences were also analyzed by epiweeks to detect significant changes in time (increase or decrease of mutation rate). Since some SARS-CoV-2 sequences in GISAID only included the sampling year or month but not the complete date (day/month/year), these were used in the global and regional analysis but not in the epiweek approach. These sequences were 1127 in E (172 from Oceania, 25 from North America, 70 from Asia, and 860 from Europe), 1136 in S (67 from Asia, 848 from Europe, 26 from North America, and 168 from Oceania), 1146 in M (70 from Asia, 873 from Europe, 26 from North America, and 177 from Oceania), and 1102 in N (50 from Asia, 853 from Europe, 25 from North America, and 174 from Oceania). The number of sequences available for each region and epiweek was not evenly distributed, especially in the last epiweeks. For this reason, and to avoid overestimation of mutation rates due to one particular country or epiweek, selected mutations were analyzed individually by country of origin and epiweek, with only epiweeks with at least 10 available sequences being considered for the time evolution analysis. For the same reason, the global analysis must be considered to be the analysis of the whole sequence dataset, taking into account the different proportions of the sequences according to the region of origin. To assess the significance of the aa changes in positions 203 and 204 of the Nucleocapsid protein, we performed an ANOVA test and Sidak test for subsequent pairwise comparisons. To assess the effects of time on the main aa combinations of these positions, we performed an exponential linear regression. Both tests were performed using Stata 16.1. using the following formula Y = b0 × (e^(b1 × X)) OR ln(Y) = ln(b0) + (b1 × X).

Location of aa changes along the protein structure domains was done according to UniProtKB (https://www.uniprot.org) and RCSB Protein Data Bank (https://www.rcsb.org) annotation.

## 3. Results

**Figure 1:**
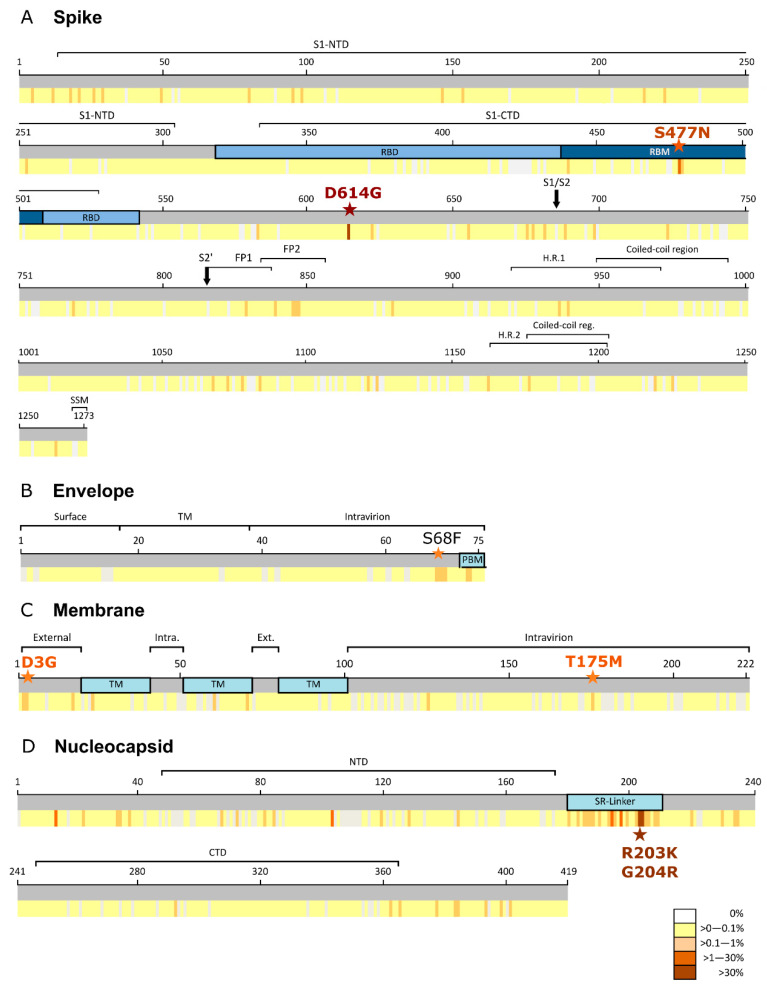
Percentage of global sequences with amino acids changes across the four SARS-CoV-2 structural proteins and their location in the protein domains. (**A**) Spike protein (1273 aa, 101,100 total sequences). With a brown star, D614G, change from aspartic acid to glycine in residue 614 of spike protein, present in 81.5% of the global sequences, and located in S1 domain, before S1/S2 furin cleavage site. With an orange star, S477N, change from serine to asparagine in residue 477, present in 4.1% of the global sequences, and located in the receptor binding motif. In light blue, receptor binding domain (RBD). In dark blue, within the RBD, receptor binding motif (RBM); (**B**) Envelope protein (75 aa, 101,376 total sequences). With an orange star, S68F, change from serine to phenylalanine at position 68 of envelope protein, present in 0.2% of the global sequences. In light blue, PDZ-binding motif (PBM); (**C**) Membrane protein (222 aa, 103,419 total sequences). With an orange star, D3G, change from aspartic acid to glycine in residue 3, and T175 M, change from threonine to methionine in residue 75. D3G and T175M were present in 0.7% and 1% global sequences, respectively. In light blue, transmembrane domains (TM); (**D**) Nucleocapsid protein (419 aa, 99,657 total sequences). With a brown star, R203K, change from arginine to lysine in position 203 and G204R change from glycine to arginine in position 204. R203K and G204R were present in 37.3% and 37% of the global sequences, respectively, and located in the serine/arginine-rich (SR)-linker. Color code as follows: white, 0% of sequences with aa changes; yellow, >0 to 0.1% of sequences with aa changes; light orange, >0.1 to 1% of sequences with aa changes; dark orange, >1 to 30% of sequences with aa changes; brown, >30% of sequences with aa changes. In light blue, serine/arginine-rich linker (SR-linker). SS, signal peptide; NTD, N-terminal domain; CTD, C-terminal domain; RBD, receptor binding domain; RBM, receptor binding motif; FP, fusion peptide; HR, heptad repeat; TM, transmembrane domain; PBM, PDZ-binding motif; SR-linker, serine/arginine-rich linker. Annotation according to UniProtKB (https://www.uniprot.org) and RCSB Protein Data Bank (https://www.rcsb.org).

A total of 105,276 worldwide SARS-CoV-2 partial and complete sequences from 117 countries were downloaded from GISAID corresponding epiweek 52, 2019 and epiweeks 1 to 37, 2020. After bioinformatics processing, we recovered and studied the genetic variability of 101,100 spike, 101,376 envelope, 103,419 membrane, and 99,675 nucleocapsid complete sequences. The epiweeks with available sequences varied across the geographic regions established according to GISAID classification (Table S1). Only Asia presented sequences in the 38 epiweeks under study, followed by Europe and North America (34 epiweeks each), Oceania (33 epiweeks), Africa (28 epiweeks), and South America (26 epiweeks). The number of analyzed sequences per protein and geographic region, the total number of mutated sequences, and the number, frequency, and nature of mutated residues in each protein are described in Table S2. The number of sequences available per country in each geographic region for each protein is listed in Table S3. The number of countries providing structural protein sequences in GISAID was 42 in Europe, 32 in Asia, 20 in Africa, 11 in North America (including Central America and the Caribbean), 9 in South America, and 3 in Oceania. Figure S1 shows the number of global and regional sequences with aa changes across S, E, M, and N SARS-CoV-2 complete structural proteins. Figure 1 shows the percentage of global sequences with aa changes for each residue across the four structural SARS-CoV-2 proteins and their location within the protein domains.

### 3.1. Spike Protein (S)

**Figure 2:**
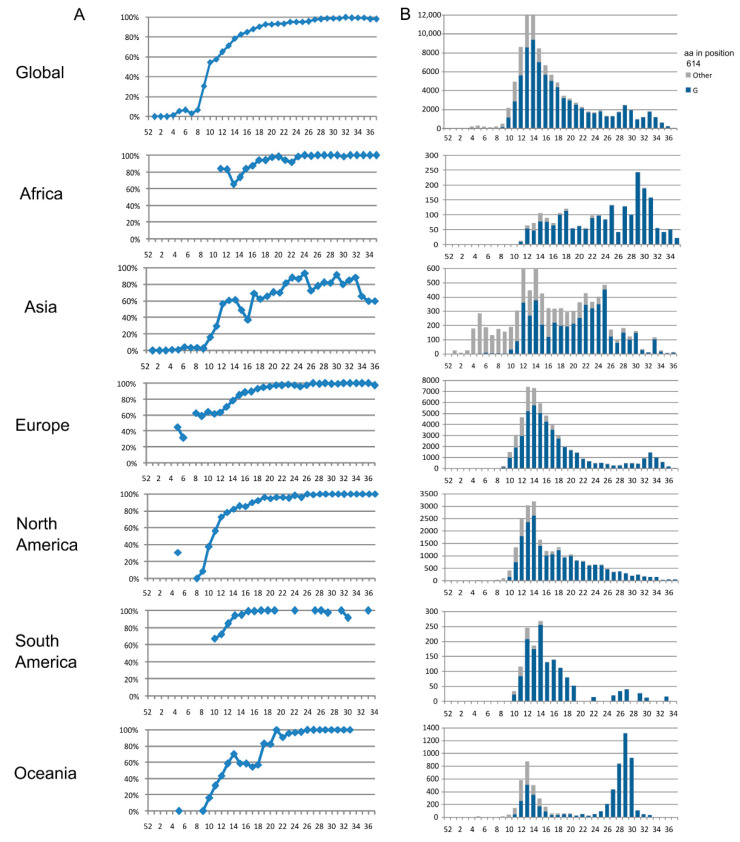
Global and regional frequency of D614G change in the spike protein over time. (**A**) Global and regional D614G frequency distribution in epidemiological weeks with, at least, 10 available sequences. The *x*-axis represents epidemiological week and the *y*-axis represents percentage of mutated sequences; (**B**) Global and regional number of sequences in spike’s residue 614 harboring aspartic acid and glycine amino acids in epidemiological weeks with at least 10 available sequences. The *x*-axis represents epidemiological week and the *y*-axis represents the number of sequences harboring other aa than G, in grey color, and G, in blue color.

Global S aa conservation was 99.97%. Among the 101,100 analyzed sequences, 2671 (2.6%) aa changes were found in 1132 (88.9%) of 1273 spike residues (Figure S1A). The most frequent aa change was D614G (81.5%, 82,183 sequences), located in S1 (Figure 1A), followed by S477N (4.1%), located in the receptor binding motif of the receptor binding domain (Figure 1A). All other substitutions had a frequency below 1%. D614G was also the most frequent aa substitution in all regions as follows: Africa (94.7%), Asia (57.7%), Europe (82.9%), North America (84.3%), South America (93%), and Oceania (82%). D614G was found for the first time in epiweek 4 in Asia in two (1%) Chinese sequences, and in Oceania in one (11%) Australian sequence. In Europe, D614G appeared in epiweek 5 for the first time, mainly in Germany (41%), and in North America (31%), in three Canadian sequences. The last regions where this mutation appeared, in epiweek 9, were Africa (in three sequences from Nigeria, Senegal, and Morocco) and South America (in three sequences from Brazil). In epiweek 10, more than half (54.7%) of the total sequences showed this change, increasing to 97.9% in epiweek 37 (Figure 2).

S477N in the S protein was present in all the geographic regions, but mainly in Oceania (56.8%, 3851 Australian sequences), where its frequency rose from 6% (epiweek 20) to 100% (epiweek 31). In the regional analysis, V1176F aa change stood out in South America (18.2%, 286 sequences), with all sequences but one belonging to Brazil.

### 3.2. Envelope Protein (E)

The global E aa conservation was 99.98%. Among the 101,376 E sequences analyzed, we found 142 aa changes in 65 (86.6%) positions of the 75 E residues (Figure S1B). All mutations were extremely infrequent, present in less than 0.3% of the total sequences (Figure 1B). The most prevalent aa change found in E was S68F, present in 221 (0.2%) global sequences, followed by L73F (122 sequences), R69I (92), P71L (68), T9I (56), and V62F (52), all with a frequency of 0.1%.

S68F was present in all regions except in South America, and mainly in Europe (86%, 177 sequences), specifically England (68.9%) where its frequency raised from epiweek 12 (0.6%) to epiweek 19 (3%), decreasing to 0.2% in the last epiweek available. This aa change first appeared in Oceania (epiweek 10), and later in Asia and Europe (epiweek 12), North America (epiweek 13), and Africa (epiweek 21). Most sequences with T9I, R69I, P71L, and L73F belonged to Europe. V62F was present in 70% of sequences from the USA in North America, where frequency increased in epiweeks 22 and 23 but dropped later.

In the analysis by geographic region, the mutation rate was less than 1% in all regions except in Africa, where V5F (1.5%) was present in 36 sequences (35 from Egypt), 92% of them belonging to the last epiweeks with African sequences available (33 and 34). In the analysis by epiweek, no steady increase over time globally or regionally was observed.

### 3.3. Membrane (M) Protein

The 103,419 M sequences analyzed presented 99.99% conservation, with 291 aa changes found in 165 (74.3%) positions of the 222 M aa (Figure S1C). Most changes had a very low frequency (≤0.2%), except for D3G (0.7%, 724 sequences), and T175M (1%, 1026 sequences) (Figure 1C). D3G was the most frequent change in Africa (3.4%) and South America (2.8%) and T175M in Europe (1.6%). However, both aa changes were present in the six geographic regions. Neither aa change showed a steady increase over time globally or regionally. D3G first appeared in European sequences (Lithuania) in epiweek 5 and was not detected in other regions until epiweek 10 (North and South America) and 11 (Asia, Africa, and Oceania). T175M was detected for the first time in epiweek 9 in Europe (England and Netherlands), and later in Asia and South America (epiweek 10), North America and Oceania (epiweek 11) and, lastly, in Africa (epiweek 12).

Globally, we observed a significant change over time in the following six aa substitutions in the M protein: A2S, L17I, D209Y, H125Y, V23L, and V60L. Change A2S increased from 0.2% in epiweek 24 to 1.4% in epiweek 30, mainly due to Australian sequences, but no further increase was observed after this epiweek. Aa change L17I increased from 0.5% in epiweek 30 to 2.8% in epiweek 32, dropping its frequency in the last epiweeks. The increase was due to English sequences. Change D209Y showed a localized increase in frequency (from 0.2% to 1.1%) in epiweek 26. This increase was mainly due to sequences from the USA, but no further increase in global or American sequences was observed after week 27. H125Y increased during the last epiweeks available, from 0.4 in epiweek 31 to 1.3% in epiweek 34, mainly due to UK sequences, specifically English and Scottish.

V23L increased from 0.4% (epiweek 19) to 1.4% (epiweek 22). This aa change was mainly present in the UK and the increase was due to sequences belonging to Wales. Lastly, V60L frequency increased around epiweeks 27 and 28 (1.6 and 1.2%) due to European sequences, specifically from England and Switzerland, decreasing later and rising again in epiweek 34 (1.4%), mainly due to sequences from Scotland and Switzerland.

### 3.4. Nucleocapsid (N) Protein

A total of 99,657 N worldwide sequences were analyzed, finding 890 aa changes in 359 (85.7%) of the 419 aa residues in the N protein (Figure S1D). The global aa conservation was 99.77%, slightly lower than the other structural proteins. Although most mutations had a low frequency, some positions showed mutations present in more than 1% of the total global sequences. It was the case for S197L (1.7%, 1686 sequences, 56% from Spain), P13L (1.8%, 1782 sequences, 62% from India and Singapore and 21% from Australia), D103Y (1.9%, 1863 sequences, 89% from England), S194L (3.2%, 3194 sequences, 39% from England and Scotland, 29% from the USA, and 11% from India), and G204R (37%, 36,598 sequences) and R203K (37.3%, 36,876 sequences) with the highest global frequency.

G204R and R203K, both located in the SR-linker (Figure 1D), tended to appear simultaneously in N protein and were the most frequent aa changes in the following six geographic regions: Africa (55.7%), Asia, (26.8%), Europe (44.1%), North America (12%), South America (60.4%), and Oceania (65.9%). The G204R and R203K combination was first detected in epiweek 5 in three German sequences, then in epiweek 8 in Nigeria, epiweek 9 in Mexico and the USA, and epiweek 10 in Asia, Oceania, and South America. The ANOVA test showed significant differences between the aa combinations in positions 203 and 204 (*p* < 0.05). When comparing pairs of possible aa combinations in these positions (Sidak test), only R203 + G204 (aa in Wuhan reference sequence NC 045512.2) and K203 + R204 (most frequent combination) showed significant differences (*p* < 0.05) with all the other present combinations (MG, KG, KL, SG, RR, IG, RV, KQ, GG, GR, KT, and NR).

**Figure 3:**
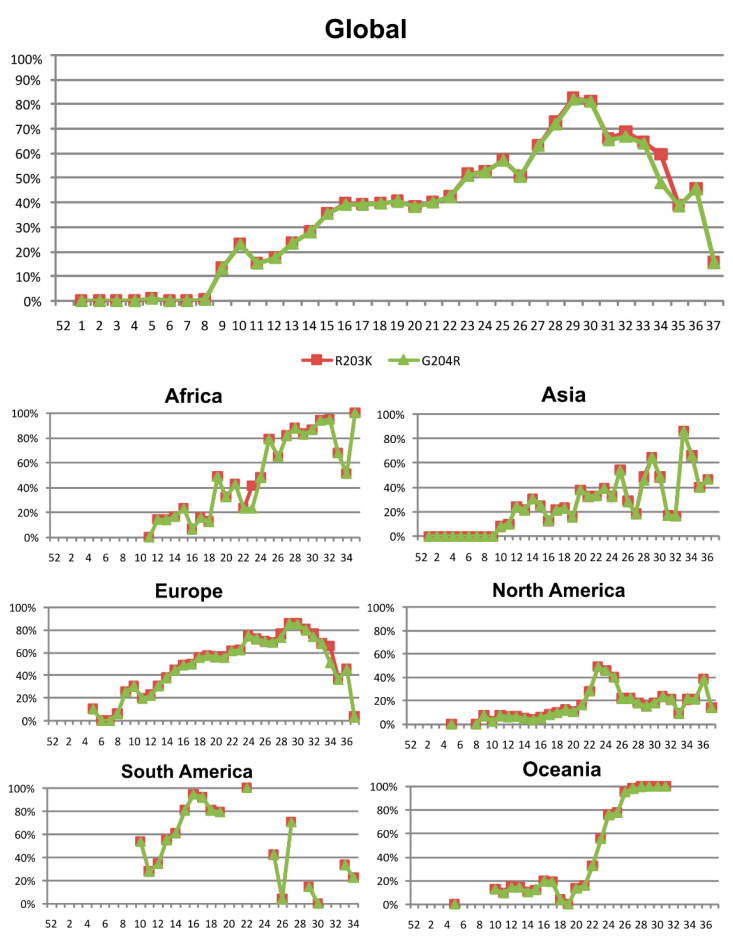
Global and regional frequency of the R203K and G204R combination in the nucleocapsid SARS-CoV-2 protein over time. The figure only includes data of those epiweeks with at least 10 nucleocapsid sequences. The *x*-axis represents epidemiological week and the *y*-axis represents percentage of mutated sequences. Color code, red (R203K) and green (G204R). R, arginine; K, lysine; G, glycine.

**Figure 4:**
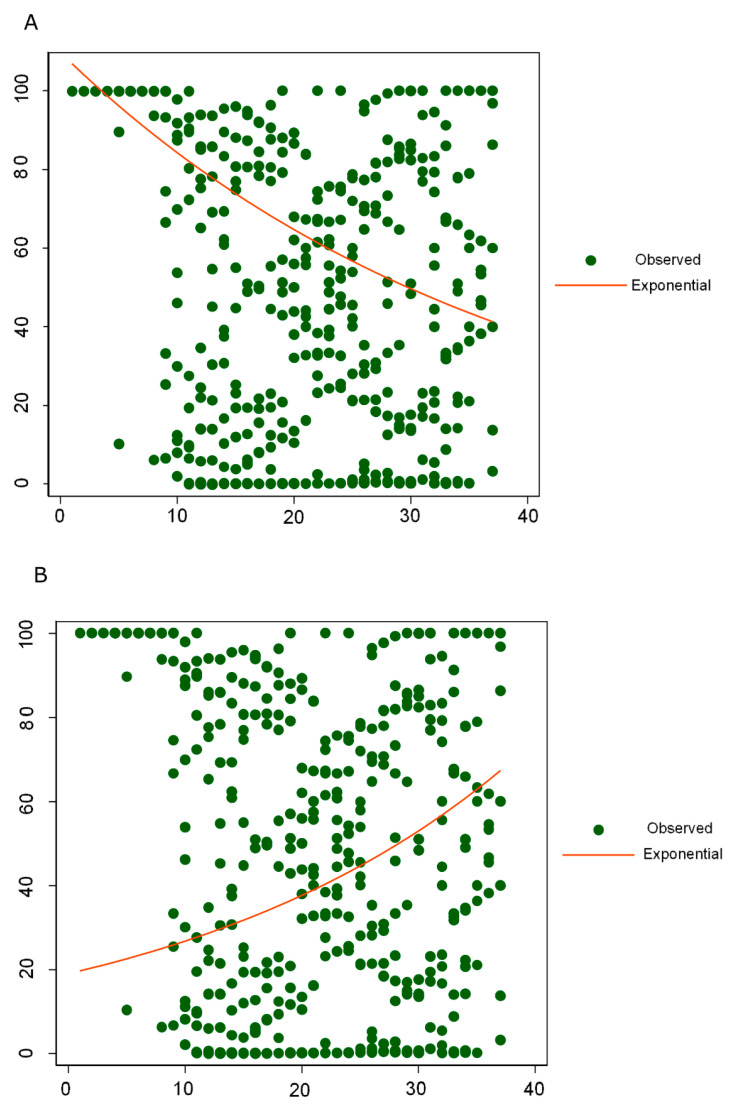
Exponential linear regression for aa combinations in positions 203 and 204 of the nucleocapsid protein. (**A**) Exponential linear regression for RG combination in positions 203 + 204 of the mucleocapsid protein. The exponential curve shows an overall decrease of the RG combination over time. b = −0.02, Y = 109.7 × (e^(−0.0264 × epiweek)) or ln(Y) = ln(109.7) + (−0.0264 × epiweek), R2 = 88.7%; (**B**) Exponential linear regression for KR combination in positions 203 + 204 of the nucleocapsid protein. The exponential curve shows an overall increase of the KR combination over time. b = 0.03, Y = 19 × (e^(0.0343 × epiweek)) or ln(Y) = ln(19) + (0.0343 × epiweek), R2 = 73.2%. The *x*-axis represents epidemiological weeks and the *y*-axis represents frequency percentage of observed combinations. Green dot, observed aa combinations in positions 203 and 204 of the nucleocapsid protein; red line, exponential curve; B, slope; R2, relative predictive power.

The global rate of the G204R and R203K combination rose from 23% in epiweek 10 to 81% in epiweek 30, dropping to 16% in epiweek 37. Figure 3 shows the occurrence of both aa changes in N by epiweek, globally, and in the six studied geographical regions. The exponential regression (Figure 4) performed in all the nucleocapsid available sequences, showed an overall decrease of RG combination over time (b = −0.02, Y = 109.7 × (e^(−0.0264 × epiweek)) or ln(Y) = ln(109.7) + (−0.0264 × epiweek), R2 = 88.7%) and an overall increase over time of KR combination (b = 0.03, Y = 19 × (e^(0.0343 × epiweek)) or ln(Y) = ln(19) + (0.0343 × epiweek), R2 = 73.2%). Nevertheless, due to the overrepresentation of European sequences in the total set of N sequences (Table S3), the regional trend of this combination showed different behaviors in the other five regions (Figure 3), with important frequency fluctuations over time. To analyze if these regional fluctuations were related to the country of origin of the sequences or the uneven distribution of the sequences from each country along the epiweeks, the statistical average of the R203K and G204R combination was analyzed between epiweeks and countries. The number and frequency of sequences carrying the R203K and G204R combination in N protein by epiweek in each geographic region and country are described in Table S4, the aa combinations for positions 203 and 204 in each region and epiweek used for the scatter plots are available in Table S5.

In North America, the frequency increased until epiweek 23 (48.7%), and then decreased until epiweek 26, stabilizing at a frequency of around 20%. As 92% of the North American N sequences belonged to the USA (Table S3), this regional curve probably describes what happened only in this country, where the R203K and G204R combination frequency reached ≈50% in epiweek 23, and then dropped to ≈20% in the following epiweeks, except for an isolated increase to 38% in epiweek 36. In Canada, the second country in this region with the most sequences, the aa combination had a median rate of 20% until epiweek 18, increasing to 73% in epiweek 21 (last epiweek with more than 10 sequences in Canada). The absence or a low number of N sequences per epiweek in the remaining North American countries excluded them for a complete similar analysis. However, when comparing data from the available epiweeks with more than 10 sequences, we also observed an increase in the frequency of the R203K and G204R combination in N sequences from Costa Rica (from 7.4% in epiweek 12 to 69.2% in epiweek 27) and Mexico (from 9.5% in epiweek 21 to 63.6% in epiweek 32); the global frequency of that combination in both countries was 28.1% and 19.3%, respectively (Table S4).

In Europe, the R203K and G204R combination steadily increased until ≈85% around epiweek 30, decreasing to 3.2% in epiweek 37, where most of the sequences belonged to Wales. Most of the European sequences (72%) belonged to the UK, mainly to England, where these aa changes increased to 89% until epiweek 31, decreasing to 57% in epiweek 36 (last epiweek that met our criteria). Similarly, in Wales, the frequency raised until epiweek 33 (91%) dropping later (4% in epiweek 37), as in Scotland (93% in epiweek 32 and 29% in epiweek 36), but not in Northern Ireland (71% in epiweek 35). Although the available N sequences differed across European countries and epiweeks, this same increase–decrease tendency was observed in Italy, Denmark, and Switzerland, whereas in other countries the R203K and G204R combination frequency increased over time (as in Netherlands, Spain, and Sweden), or only in the last available epiweeks with 10 sequences (as in Germany). A steady increase in frequency was observed in Portugal, France, and Russia, whereas in Austria the frequency remained stable and in Belgium, it varied greatly without a clear tendency (Table S4).

In Africa, the frequency increased from 14% (epiweek 12) to 94.5% (epiweek 32), although a drop in frequency was observed in epiweeks 33 and 34, where only sequences from South Africa and Egypt were available. Most African sequences belonged to South Africa (61%), followed by the DRC (12%), Egypt (7%), and Senegal (5%) (Table S3). South Africa showed a steady increase in the R203K and G204R combination frequency (≈90% frequency in epiweeks 30–35), in the DRC the frequency varied largely between epiweeks, and in Egypt it was very infrequent, only present in 4% of the total sequences, explaining the drop of the regional rate (Table S4).

In Oceania, the R203K and G204R combination frequency steadily increased from epiweek 20–31. Of note, 96% of Oceania’s sequences belonged to Australia where this combination was present in ≈90% sequences since epiweek 26. In New Zealand, it increased from 6% (epiweek 12) to 53% (epiweek 17), the only epiweeks with enough sequences for analysis (≥10), although its total frequency was lower than in Australia (11.8% vs. 68%) (Table S4).

In Asia, the combination increased over time, except for epiweeks 26 and 27 and 31 and 32 where the frequency dropped. Most Asian sequences in these epiweeks (>50% in epiweek 26 and 100% in epiweeks 27 and 31) were from Singapore and South Korea, where the R203K and G204R combination was infrequent, explaining the frequency drop in these epiweeks. More than half of Asia’s sequences were from India (31.3%), China (12.7%), and Singapore (11.5%) (Table S3). The regional presence of the R203K and G204R combination changes in N varied greatly between countries (Table S4). Considering those countries with >100 sequences, the higher frequencies were found in Bangladesh (87%), and Oman (75%), and the lowest in Malaysia (6%), Singapore (8%), China (9%), and South Korea and Thailand (10%). In India (total rate 36%), this combination frequency slowly increased until epiweek 25 (56%), dropping to ≈20% in the following epiweeks, and rising again to >80% in the last epiweeks that met our criteria (epiweeks 29, 33, and 34). In China, although the R203K and G204R combination total frequency was low, most sequences grouped up in the first epiweeks, where these changes were absent or extremely infrequent. However, in epiweek 14 (10 sequences) the frequency reached 40%, and in epiweeks 28–30 (next epiweeks that meet our criteria) this frequency increased to >80%, as happened in India. In Singapore, the R203K and G204R combination was absent until epiweek 28, when it increased from 4% to 47% in epiweek 36.

Finally, in South America, although some epiweeks did not meet our criteria for the time analysis, the R203K and G204R combination in N increased until epiweek 22, with great variations in frequency in the last epiweeks. Of note, the bulk of the sequences available belonged to Brazil (52%), followed by Colombia (13%), Chile (12%), and Peru (8%) (Table S3). Brazil showed the highest frequency of this combination (88.7%, Table S4), and in epiweeks 10–19 (those that met our criteria), the R203K and G204R combination raised >90% since epiweek 15, observing a drop in the next epiweeks with <10 sequences, with the absence of that combination in the nine N sequences available in epiweek 30. In Colombia, Chile, and Peru, an increasing tendency was also observed in epiweeks meeting criteria (with ≥10 sequences). The observed increase was from 13.6% (epiweek 12) to 18.2% (epiweek 15) in Colombia, from 24% (epiweek 11) to 67% (epiweek 18) in Chile, and from 37% (epiweek 12) to 79.2% (epiweek 27) in Peru. In South America, in epiweeks 26 (3.6% regional rate) and 29 (14.3%), most of the available sequences belonged to Suriname, where these changes were extremely infrequent (6.67% total frequency), impacting in a low regional rate in these epiweeks.

In the regional analysis, the most frequent N mutations were the already mentioned D103Y, S194L, and S197L in Europe (3.2%, 2.8%, and 2.3%, respectively), S194L in North America (4.3%); P13L and S194L in Asia (15% and 6.4%), and P13L and S197L in Oceania (5.7% and 4.8%, respectively). Other frequent mutations not mentioned before were S202N in Asia and Africa (2.5% and 3.8%, respectively), I292T in South America (25%), Q384H in Africa (7%), and L230F in Oceania (2.7%).

## 4. Discussion

Monitoring SARS-CoV-2 genetic diversity and emerging mutations in this ongoing pandemic is crucial for understanding the evolution of this new coronavirus and assuring the performance of new diagnostic tests, vaccines, and therapies against COVID-19. This descriptive study analyzes the aa variability of the four SARS-CoV-2 structural proteins in the largest set of worldwide sequences for over 9 months since the beginning of the pandemic, describing the most frequent aa changes and their evolution in time by geographic region and epiweek.

We observed that, despite the extremely high conservation of SARS-CoV-2 structural proteins (99.99% in M, 99.98% E, 99.97% S, and 99.77% N), all proteins presented mutated residues across isolates, with different temporal trends. The S protein harbored the highest proportion of aa positions admitting changes along its sequence (88.9% of 1273 aa), followed by the E protein (86.6% of 75 aa), N protein (85.7% of 419 aa), and the M protein (74.3% of 222 aa), most of them presenting extremely infrequent changes.

In the S protein, the most remarkable aa change was D614G, present in eight out of 10 global spike analyzed sequences. This mutation has been increasing its rate over time both globally and regionally, spreading in all of the six studied regions and establishing as the dominant polymorphism. However, any aa change frequency taking place in the last epiweeks should be interpreted with caution, since it can be due to the low number of SARS-CoV-2 spike sequences for advanced epiweeks in the study sequence set, impacting the regional data. D614G has been previously reported in several studies, some of them also including a large number of sequences [25,33], but its biological impact has not been elucidated yet.

Some reports suggest that D614G spread could be explained by a founder effect, without the need for a selective advantage, especially at the beginning of an epidemic, when most individuals are susceptible [25]. However, other reports hypothesize that D614G enhances viral fitness [33,34]. It has been associated with lower RT-PCR cycle thresholds, and therefore greater infectivity [33], as well as infecting ACE2-expressing cells more efficiently in vitro, showing greater transmissibility [34]. A recent study has reported that D614G increases infectivity in human lung cells or cells with bat or pangolin ACE2 receptor in cell cultures, and that D614G shifts the S protein conformation toward an ACE2-binding fusion-competent state [35].

Previous reports hold that the E and M proteins are conserved across ß-coronaviruses, showing high structural similarity to pangolin and bat CoVs isolates [36]. Despite nearly identical conservation (99.98% vs. 99.99%), E harbored fewer aa changes than M (142 changes in 65 of 75 E residues vs. 291 changes in 165 of 222 M residues), S68F being the most prevalent, in only 0.2% of the global sequences. The CoVs E secondary structure consists of a short (7–12 aa), hydrophilic N-terminal domain (NTD), a large hydrophobic transmembrane domain (25 aa) with a large proportion of valine and leucine, and a hydrophilic C-terminal domain (CTD) [13]. Although infrequent, most mutations with a global rate ≥0.1% were found in the CTD, including S68F. The last four aa of CoVs CTD harbor a post-synaptic density protein-95/discs large/zonula occludens-1 (PDZ)-binding motif (PBM) that has been reported as a major determinant of virulence in SARS-CoV [13,37]. The PBM is essential for the SARS-CoV virus, as mutation or deletion of PBM or full-length E causes revertant mutants with PBM restoration [38]. Of note, SARS-CoV-2 reference sequence harbors in the PBM the same four aa (D72, L73, L74, and V75) as the SARS-CoV reference isolate (NCBI accession number NC_004718.3). In this study, we found only 221 sequences of the total 101,376 E sequences (0.2%) with aa changes in the PBM, being L73F the most prevalent, and with no frequency increase over time. Further studies are required to establish the effect of C-terminal and PBM mutations in SARS-CoV-2 E protein structure and protein–protein interactions, as well as future temporal analysis to detect any increase in their frequency.

The M protein was the most conserved protein (99.99%), but it also presented aa changes in the global approach, D3G and T175M being the most prevalent of the 291 aa changes found. The M protein consists of a short NTD ectodomain, three transmembrane domains, and a long CTD situated inside the virion particle [39]. The D3G mutation belongs to the exposed NTD, whereas T175M is located in the CTD. It has been stated that mutations in NTD could play an important role in host–cell interaction [36]. Of note, glycine in position 3 has been also found in bat and pangolin CoVs [36].

The N protein showed the lowest conservation rate (99.77%), presenting 890 changes in 359 of 419 N residues in the analyzed sequence set. This protein is divided into two main domains, i.e., the N-terminal RNA-binding domain (NTD) and the C-terminal dimerization domain (CTD), divided by a central serine/arginine-rich (SR)-linker responsible for phosphorylation [16,17,40]. Phosphorylation of this SR-link motif in SARS-CoV modulates nucleocapsid multimerization, translational inhibitory activity and cellular localization [41] The main finding in this protein was the simultaneous increase in the frequency of both G204R and R203K, the most frequent mutations in the N protein (37% and 37.3%, respectively) at the global and regional levels. Although the K203 and R204 combination showed a globally increasing incidence as compared with the R203 and G204 combination (aa in Wuhan reference sequence NC 045512.2), this behavior could have been distorted by the large number of global sequences belonging to Europe in the studied sequence set. When analyzing each geographical region individually, different temporal trends of the G204R + R203K pair were observed according to the country of origin of the N sequences and even between epiweeks. In Oceania and Africa, the rate seemed to be increasing, although not enough sequences were available for the last epiweeks’ analysis. In most European countries, the G204R and R203K combination seemed to have steadily increased in time during the beginning of the pandemic, however, this upward trend may be disappearing in the last epiweeks for some countries. Further analysis should be conducted in the following epiweeks to check the incidence of these mutations in each country and geographic region with new and recent sequences when available.

G204R and R203K are located in the SR-linker of the N protein (aa 180–210), as well as the other two aa changes found (S194L and S197L) with >1% global rate. Therefore, the SR-linker was the most variable region within the N protein, with a median substitution rate of 2.7% vs. 0.03% in CTD and 0.04% in NTD. The SR-linker forms a phosphorylation-dependent binding domain for protein 14-3-3, a signaling molecule involved in various cellular processes, such as cell cycle, survival, and death [42]. It has been reported that mutations in this region could have an important biological impact, including aa change S197L, present in 1686 N sequences in our study, and representing 1.7% of the complete sequence set. S197L is a phosphorylation site for kinases involved in the control of the cell cycle. Furthermore, a change in this position would also affect the adjacent residue T198, another phosphorylation site [42]. A recent study suggested that the co-occuring mutations R203K and G204R may decrease the overall structural flexibility of SARS-COV-2 N protein [43]. Further analysis is needed to evaluate the impact of N SR-linker mutations in SARS-CoV-2 transmissibility and virulence, as well as of the observed global increase of the G204R and R203K combination.

The main limitation of the temporal analysis performed at the regional level is the uneven country and epiweek distribution of available sequences, mainly in Europe (with a large set of sequences belonging to England), North America (mostly sequences from the USA), and South America (with most sequences belonging to Brazil). In fact, in some countries, none or just a few sequences are available in the GISAID database, as we reported in Table S3. Moreover, in some regions, none or just a few SARS-CoV-2 sequences were available for advanced epiweeks, impacting the regional data. However, these limitations could be easily overcome by periodically checking the GISAID database as more sequences are uploaded daily, and therefore reinforce economic support for viral sequencing in countries. Therefore, we encourage worldwide upload of SARS-CoV-2 genomic sequences in this widely used database and continuous analysis of their variability in order to be able to detect any emerging mutation early.

Although SARS-CoV-2 presents a mutation rate around 10 times lower than for other RNA viruses (around 33 genomic mutations per year) [44], this virus still acquires some mutations as it spreads from host to host, as our presented genomic data support. As more data is made available during the pandemic, we expect this study will facilitate ongoing investigation of SARS-CoV-2 variability and its consequences in COVID-19 evolution. Further analysis of linked mutations, virus-host protein interactions, and protein structure are crucial to understand the global and regional implications of the aa changes previously described. The presented data provide useful knowledge for future diagnostic, therapeutic, or vaccination approaches directed to these structural SARS-CoV-2 proteins, which could help disease control and prevention efforts, and for a better understanding of how this virus expands in certain countries or geographic areas.

## Data Availability

All the sequences dataset used in this study are available in the public GISAID database (https://www.gisaid.org). All data regarding results are available in the supplementary information.

## Supplementary Materials

The following are available online at https://www.mdpi.com/1999-4915/13/2/243/s1, Figure S1: Number of global and regional sequences with amino acid changes across SARS-CoV-2 structural proteins. (**A**) Spike protein (1273 aa, 101,100 total sequences). With an asterisk, D614G, change from aspartic acid to glycine in residue 614 of spike protein. D614G was the most prevalent aa substitution present in 82,183 (81.5%) of the global sequences; (**B**) Envelope protein (75 aa, 101,376 total sequences). With an asterisk, S68F, change from serine to phenylalanine at position 68 of envelope protein. S68F was the most prevalent change found, present in 221 (0.2%) global sequences; (**C**) Membrane protein (222 aa, 103,419 total sequences). With an asterisk, D3G, change from aspartic acid to glycine in residue 3 and T175 M, change from threonine to methionine in residue 75. D3G and T175M were present in 724 (0.7%) and 1026 (1%) global sequences, respectively; (**D**) Nucleocapsid protein (419 aa, 99,657 total sequences). With an asterisk, R203K, change from arginine to lysine in position 203 and G204R change from glycine to arginine in position 204. R203K and G204R were present in 36,876 (37.3%) and 36,598 (37%) global sequences, respectively. Color code, red (global), green (Africa), purple (Asia), dark blue (Europe), orange (North America), light blue (South America), pink (Oceania). D, aspartic acid; G, glycine; S, serine; F, phenylalanine; T, threonine, M, methionine; R, arginine; K, lysine, Table S1: Epiweeks with available sequences in each structural SARS-CoV-2 protein across geographic regions. Epiweek, epidemiological week; S, spike protein; E, envelope protein; M, membrane protein; N, nucleocapsid protein, Table S2: Analyzed sequences, number of aa changes, and mutated aa positions in each SARS-CoV-2 structural protein by geographic region. S, spike protein; E, envelope protein; M, membrane protein; N, nucleocapsid protein, Table S3: Available sequences per country and SARS-CoV-2 structural proteins. S, Spike protein; E, envelope protein; M, membrane protein; N, nucleocapsid protein, Table S4: Number and frequency of sequences carrying R203K and G204R aa change combination in N protein by epiweek in each geographic region and country. Epiweek, epidemiological week; N, nucleocapsid protein; N.A., North America; S.A., South America. Table S5: Amino acid combinations in positions 203 and 204 of the Nucleocapsid protein according to region and epiweek.
