# Supplementary figures and images for: Evolution of SARS-CoV-2 Envelope, Membrane, Nucleocapsid, and Spike Structural Proteins from the Beginning of the Pandemic to September 2020: A Global and Regional Approach by Epidemiological Week
Source: Viruses. 2021 Feb 4;13(2):243. doi: 10.3390/v13020243 (PMC7913946; doi:10.3390/v13020243)

1    **Supplementary Figure 1.**

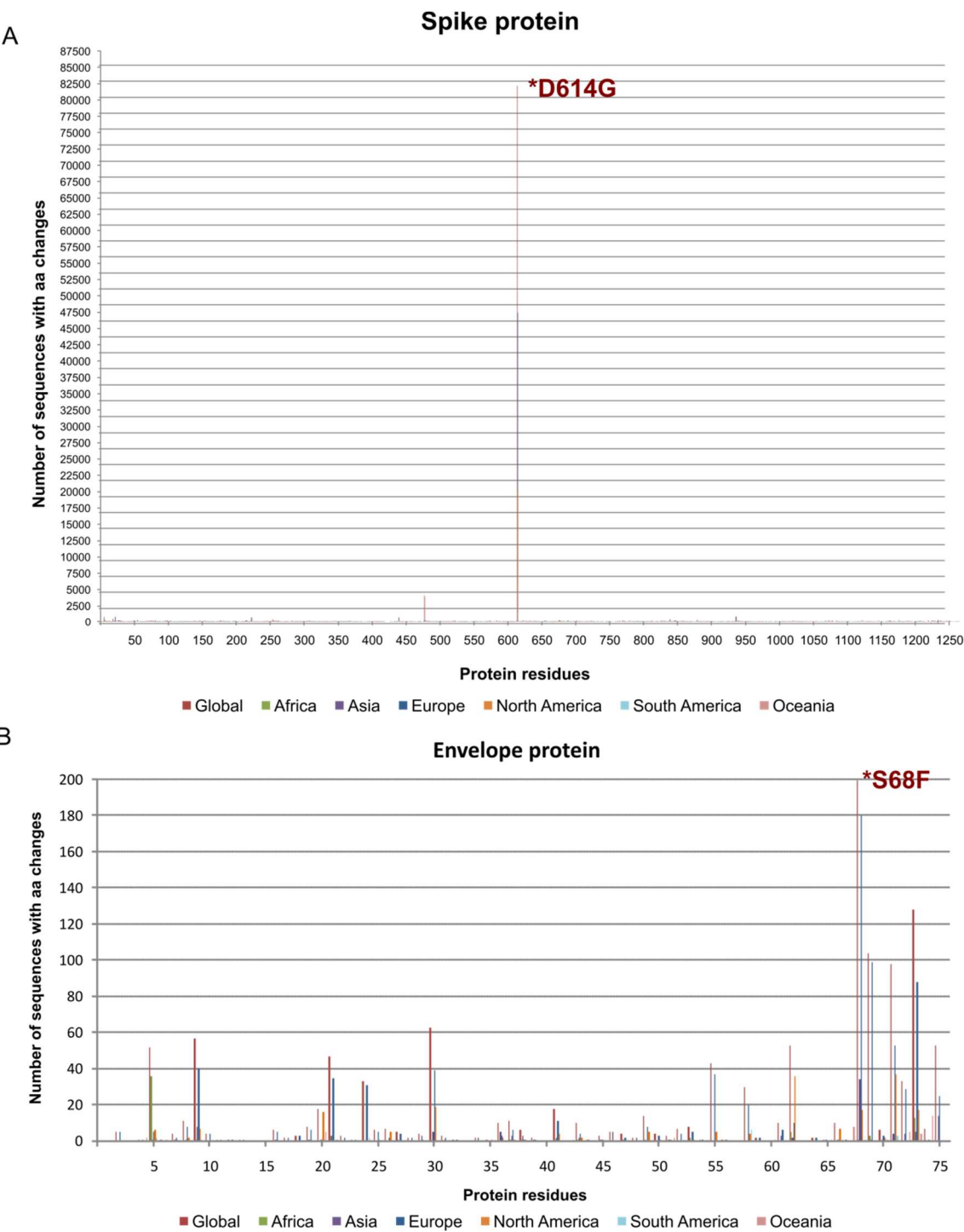

2

3

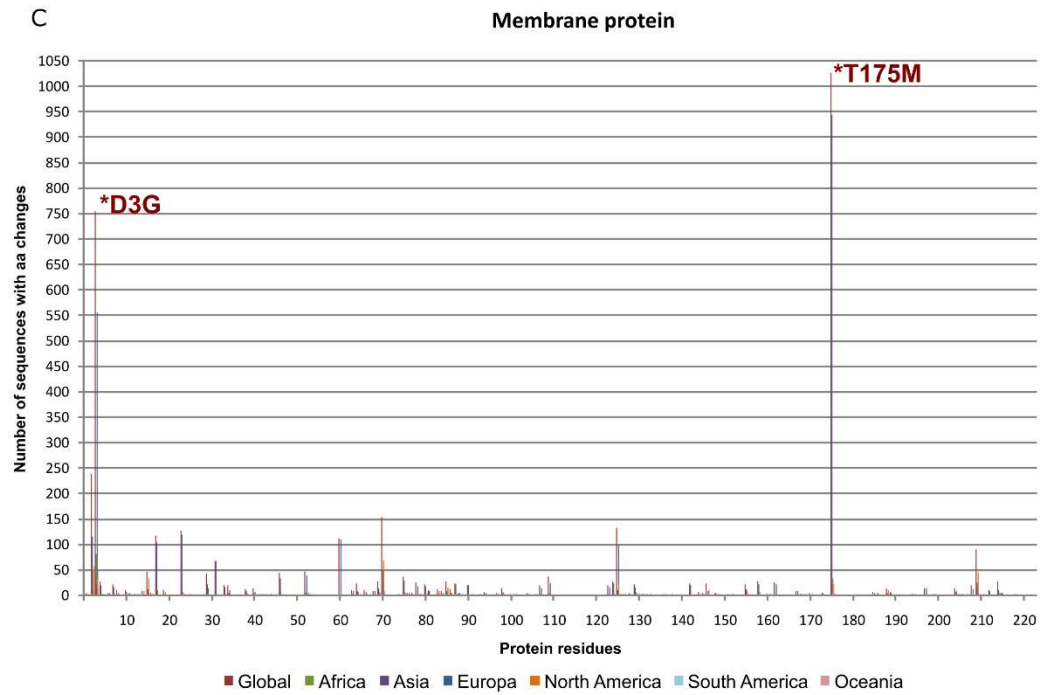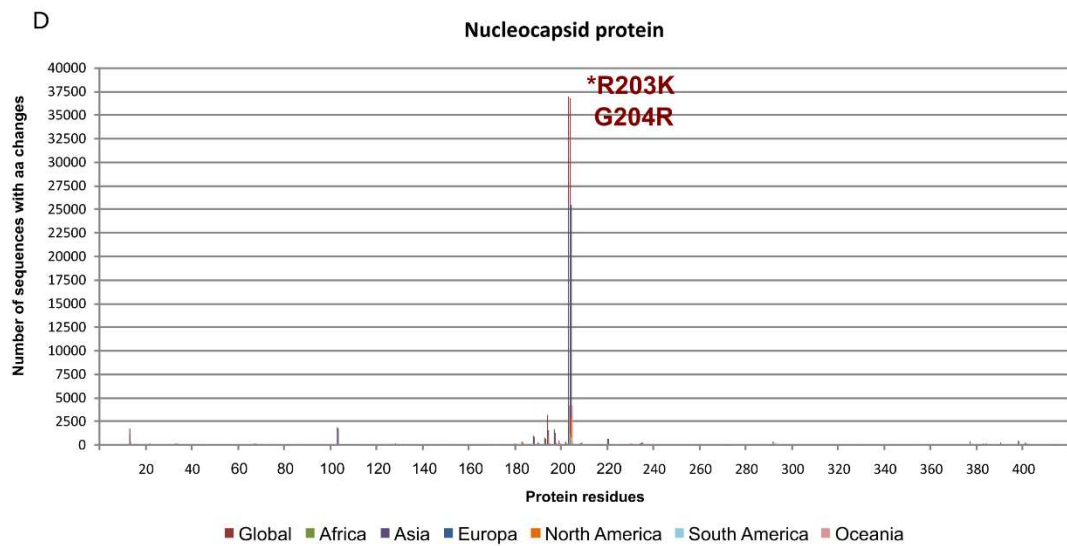

Supplement: Supplementary file 1 [file viruses-13-00243-s001.zip › Supplementary Figure 1.pdf]
